# Impact of enteropathogens on faltering growth in a resource-limited setting

**DOI:** 10.3389/fnut.2022.1081833

**Published:** 2023-01-10

**Authors:** Furqan Kabir, Junaid Iqbal, Zehra Jamil, Najeeha Talat Iqbal, Indika Mallawaarachchi, Fatima Aziz, Adil Kalam, Sahrish Muneer, Aneeta Hotwani, Sheraz Ahmed, Fayaz Umrani, Sana Syed, Kamran Sadiq, Jennie Z. Ma, Sean R. Moore, Asad Ali

**Affiliations:** ^1^Department of Pediatrics and Child Health, The Aga Khan University, Karachi, Pakistan; ^2^Department of Biological and Biomedical Sciences, The Aga Khan University, Karachi, Pakistan; ^3^Department of Public Health Sciences, University of Virginia, Charlottesville, VA, United States; ^4^Division of Pediatric Gastroenterology, Hepatology, and Nutrition, Department of Pediatrics, University of Virginia, Charlottesville, VA, United States

**Keywords:** environmental enteric dysfunction (EED), enteropathogen, growth faltering, TaqMan array card (TAC), *Giardia*, biomarkers

## Abstract

**Introduction:**

Environmental enteropathy is an important contributor to childhood malnutrition in the developing world. Chronic exposure to fecal pathogens leads to alteration in intestinal structure and function, resulting in impaired gut immune function, malabsorption, and growth faltering leading to environmental enteropathy.

**Methods:**

A community-based intervention study was carried out on children till 24 months of age in Matiari district, Pakistan. Blood and fecal specimens were collected from the enrolled children aged 3–6 and 9 months. A real-time PCR-based TaqMan array card (TAC) was used to detect enteropathogens.

**Results:**

*Giardia*, *Campylobacter* spp., enteroaggregative *Escherichia coli* (EAEC), Enteropathogenic *Escherichia coli* (EPEC), Enterotoxigenic *Escherichia coli* (ETEC), and *Cryptosporidium* spp. were the most prevailing enteropathogens in terms of overall positivity at both time points. Detection of protozoa at enrollment and 9 months was negatively correlated with rate of change in height-for-age Z (ΔHAZ) scores during the first and second years of life. A positive association was found between Giardia, fecal lipocalin (LCN), and alpha 1-Acid Glycoprotein (AGP), while Campylobacter spp. showed positive associations with neopterin (NEO) and myeloperoxidase (MPO).

**Conclusion:**

Protozoal colonization is associated with a decline in linear growth velocity during the first 2 years of life in children living in Environmental enteric dysfunction (EED) endemic settings. Mechanistic studies exploring the role of cumulative microbial colonization, their adaptations to undernutrition, and their influence on gut homeostasis are required to understand symptomatic enteropathogen-induced growth faltering.

## Introduction

Environmental enteric dysfunction is considered one of the major contributors to malnutrition in children living in resource-limited settings. In the presence of inadequate diet and limited access to clean water and sanitation, exposure to enteropathogens leads to intestinal injury and compromised nutritional absorption with or without overt diarrhea (1). These features collectively contribute to EED, associated with stunting, impaired oral vaccine response, diminished neurodevelopment, and metabolic consequence (2, 3). Current literature suggests that convergence of nutritional deficiencies and polymicrobial exposure invokes detrimental effects on the linear development of young children with an increased risk of under-five mortality, especially from infectious disease (4). Recurrent episodes of infectious diarrhea have been associated with growth faltering, yet a decline in diarrheal rates failed to reduce the prevalence of stunting (5). Rotavirus, *Campylobacter* spp., *Shigella*, heat-stable toxin-producing enterotoxigenic *Escherichia coli*, *Cryptosporidium* spp., and Norovirus have been reported to exhibit the highest attributable burdens of diarrhea (3, 6). However, same pathogens were also reported in non-diarrheal stools of children living in resource-limited settings (6, 7). These findings led to exploring “asymptomatic” enteric infections without overt diarrhea with growth faltering, especially in high malnutrition settings.

This asymptomatic colonization may cause intestinal inflammation with or without diminished barrier and absorptive functions of the gut. Moreover, enteropathogen colonization is reported to exhibit an association with higher inflammatory markers in the blood (C-reactive protein, AGP, etc.) and fecal markers of intestinal inflammation such as neopterin (NEO), myeloperoxidase (MPO) (8). Intestinal microbial alterations and metabolic perturbations, in addition to genetics, have also been associated with poor growth in these children (9).

While investigating novel therapeutic strategies that may reverse damage resulting from nutritional deficiencies and microbial insults, it is important to understand the role of subclinical intestinal co-pathogen colonization and intestinal dysfunction that may lead to the compromised growth rate in children (10). Adapting to the ongoing damage caused by these pathogens could hinder nutritional interventions and anti-inflammatory-based therapies. Even antibacterial therapies have shown mixed effects (11), as protozoa and subclinical viral colonization have also been reported in the context of EED (7). This study focuses on the prevalence of asymptomatic enteric co-pathogen colonization and their association with growth rate and inflammatory biomarkers in children living in high malnutrition settings to explore more relevant microbes to EED.

## Materials and methods

### Study design and participants

The “Study of environmental enteropathy and malnutrition (SEEM)” was a community-based interventional study in which active surveillance of enrolled newborns was carried out until 24 months of age. The study duration was April 2016 to April 2018. Matiari district holds a relatively high burden of maternal and childhood illness as indicated by significantly high ratios of maternal mortality of 259/100,000 live births, neonatal mortality of 46.9/1000 live births, and stillbirth rates of 42.8/1000 total births (12).

A detailed protocol of the current study has been published (13). Children were recruited after 6 months of follow-up with at least two anthropometries as per study criteria by our community health workers (CHW). Eligibility criteria included newborns aged up to 14 days without any major congenital abnormalities or disease and whose parents willingly consented to participate in the study. Children whose families were planning to move out of the study area within 6 months of recruitment were excluded from the study. Follow-up of enrolled children for up to 24 months with weekly home visits by the CHWs. At each weekly visit, CHWs obtained morbidity data, including the number of days of diarrhea in the preceding week.

Furthermore, children recorded anthropometry measurements, including height (1 mm precision using a rigid length board with a movable foot piece) and weight (20 g precision electronic scale; TANITA 1584), on a monthly basis. The anthropometric analysis was done by standard WHO Software (ENA smart 2011, Emergency Nutrition Assessment). Anthropometry data collected between 3 and 6 months was used to calculate weight-for-height Z (WHZ) and height-for-age Z (HAZ) scores based on World Health Organization (WHO) growth reference standards.

### Sample collection

Blood and fecal specimens were collected from the enrolled children at 3–6 and 9 months of age to detect EED-associated biomarkers. A total of 1–2 ml of blood was collected in a gel-top tube (BD vacutainer) by venipuncture, and serum was separated within 2 h of blood collection. Fecal samples were collected by using stool kits. Specimens were transported daily at 2–4°C from Matiari field lab to Infectious Diseases Research Laboratory (IDRL), Karachi. All samples were stored at −80°C until further processed.

### TaqMan array card (TAC) assay for fecal analysis

Fecal samples were analyzed using TAC assay to detect enteropathogens in a stool sample. Briefly, stool samples underwent lysate preparation followed by mechanical disruption through bead-beating, removal of inhibitors, purification, and elution of TNA using QIAamp Fast Stool DNA Mini kit (Qiagen, Valencia, CA, USA) according to the manufacturer’s instructions. Briefly, 180–220 mg of stool samples were spiked with InhibitEX Buffer containing Phocine Herpes Virus (PhHV; Houpt Laboratory, UVA, Charlottesville, VA, USA) and MS2 (MS2 bacteriophage; Houpt Laboratory, UVA, Charlottesville, VA, USA) extrinsic controls as DNA and RNA targets, respectively, for validation and monitoring of the efficiency of extraction, reverse transcription, and amplification steps. Samples were homogenized, bead-beaten for 2 min with ∼370 mg of glass beads (Sigma, St. Louis, MO, USA), and incubated at 95°C for 5 min. Samples were further centrifuged at 14,000 rpm for 1 min to pellet any stool particles, then 600 μl of InhibitEx lysate were extracted and eluted in 200 μl of elution buffer.

TaqMan array card protocol was performed as described previously (14). Briefly, 20 μl of TNA was added in 80 μl of Agpath one-step RT PCR master mix which contained nuclease-free water, 2X Agpath buffer, and enzyme (Thermo Fisher Scientific, USA). After thorough mixing, the reaction mix was loaded onto the TAC card and centrifuged twice at 1,200 rpm for 1 min. The card was sealed, followed by excision of loading ports, and then the card was inserted into QuantStudio 7 Flex platform (Applied Biosystems, Thermo Fisher Scientific). The cycling conditions were as follows: 45°C for 20 min and 95°C for 10 min, followed by 40 cycles of 95°C for 15 s and 60°C for 1 min. A total of eight samples were run on a single card, extraction blanks (consisting of nuclease-free water, spiked with PhHV and MS2) and PCR blanks (consisting of nuclease-free water only) were also run after every third card to rule out false positivity/negativity aspects. The sample was considered valid positive if 1) the sample’s target Ct value was less than 35.0, 2) the reference extraction blank was negative for each target, and 3) the internal controls (PhHV, MS2) had a Ct value less than 35.0. Our TAC cards were customized to detect common enteropathogens and antimicrobial-resistant (AMR) genes such as *bla* ctx-M-1-2-9, *bla* ctx-M-8-25, and *mphA*.

### Measurement of biomarkers

Stool inflammatory biomarkers such as MPO, LCN and NEO, were detected in fecal samples. In contrast, systemic biomarkers included leptin, ferritin, insulin-like growth factor-1 (IGF-1), C- reactive protein (CRP), alpha 1-Acid Glycoprotein (AGP), and glucagon-like peptide 2 (GLP-2) were detected in serum samples. For serum separation, the blood tube was centrifuged at 4,000 rpm for 10 min, aliquoted in small volumes, and stored until testing. Commercial ELISA kits were used for the detection of MPO (Immunodiagnostic AG, Stubenwald-Allee, and Bensheim), NEO (GenWay Biotech, San Diego, CA, USA), GLP-2 (USCN, Life sciences Inc, Wuhan, China), and Reg1B (TechLab, Blacksburg, Virginia). The Regenerating Family Member 1 Beta (Reg1B) kits were kindly provided by the Bill Petri’s Lab at the University of Virginia (UVA) for blood and fecal Reg1B estimation. For measuring leptin in blood, ELISA kits by Quantikine ELISA, Human leptin Immunoassay, Catalog Number DLP00, and R&D Systems were utilized. The final dilution of serum and fecal biomarkers was determined by the selection of the most suitable concentration falling in the standard curve’s linear range., NEO at the dilution of 1:250 and MPO at 1:500. All plates were read on Biorad iMark (Hercules, CA, USA) plate reader. DuoSet ELISA DY1757 was used to measure natural and recombinant human Lipocalin-2. Analysis of CRP, ferritin and AGP was done on Hitachi 902 analyzer (Roche Diagnostics, Holliston, MA, USA) by Immunoturbidimetric assay, and IGF-1 was measured on LIAISON [Diasorin Saluggia (VC) Italy].

### Statistical analysis

Continuous variables were summarized as mean (SD) or median (Q1, Q3), where Q1 is denoted as 1st quartile and Q3 as the 3rd quartile. Categorical variables were summarized using frequency and percentages. Continuous variables were compared between binary groups using Wilcoxon rank-sum test and among multicategory variables using the Kruskal Wallis test. Categorical variables were compared between groups using Fisher’s exact test. *Post hoc* comparisons were made for significant variables using Sidak multiple comparisons adjustment, which controls familywise error rate (FWER). The enigmatic associations of biomarkers and enteropathogens were assessed using quantitative Spearman correlation.

### Ethics statement

This study was approved by the Ethical Review Committee (ERC) of The Aga Khan University in 2015 (3836-Ped-ERC-15). Consent forms were obtained from the parents or caretakers of enrolled children. All experiments on Human Subject Research were conducted according to the relevant guidelines and regulations.

## Results

The demographics and baseline characteristics of the study participants are summarized in Table 1.

**TABLE 1:** Characteristics of the study group.

Factors	*n* = 416	
**Gender**		
Male (*N*, %)	250 (60.10%)	
Female (*N*, %)	166 (39.90%)	
Gestational age (wks), mean (SD)	38.72 (1.06)	
HAZ at enrollment	−1.61 (−2.41, −0.87)	
ΔHAZ by 12 months	−0.55 (−1.43, 0.10)	
ΔHAZ by 24 months	−0.69 (−1.46, 0.04)	
WAZ at enrollment	−1.88 (−2.76, −1.16)	
ΔWAZ by 12 months	−0.49 (−1.21, 0.44)	
ΔWAZ by 24 months	−0.42 (−1.07, 0.51)	
Diarrheal episodes	13.00 (7.00, 18.00)	
**Fecal biomarkers**	**3–6 months**	**9 months**
Myeloperoxidases (ng/ml)	7697 (2700, 18165)	3742.75 (1531, 9850)
Lipocalin (ng/gm)	15570 (10580, 23809)	21852 (13518, 31576)
Neopterin (nmol/L)	1800 (836, 2725)	1862.5 (919, 2775)
**Systemic biomarkers**	**3–6 months**	**9 months**
Leptin (pg/ml)	157.4 (80.5, 281.2)	181.19 (102.5, 293.8)
Ferritin (ng/ml)	82.0 (33.0, 176.0)	17.75 (7.0, 37.0)
IGF (ng/mL)	23.31 (14.1, 39.2)	20.3 (12.4, 32.7)
AGP (mg/liter)	20.25 (12.4, 32.7)	101.6 (77.0, 135.9)
CRP (mg/l)	0.14 (0.05, 0.39)	0.16 (0.06, 0.41)
GLP (pg/ml)	1016.7 (719.5, 1555.0)	1208.9 (815.2, 1760.5)

Continuous data are expressed as mean (SD) or median (Q1, Q3) unless specified; Q1: 1st quartile; Q3: 3rd quartile; SD: standard deviation.

### Protozoal colonization of fecal samples is associated with a decline in ΔHAZ

We stratified enteropathogens into three categories as bacterial, viral, and protozoal targets. Bacterial targets were detected most frequently (75%) in 3–6 months’ fecal samples, followed by protozoal (50.7%) and viral targets (47.8%), as shown in Supplementary Table 1. A similar trend was observed in 9 months’ fecal samples with a 78.6% abundance of bacterial targets, followed by protozoal (58.9%) and viral targets (53.5%). The presence of protozoa at 6 months was negatively correlated with ΔHAZ at 12 months of age (*p* = 0.011) and 24 months (*p* = 0.004) as shown in Table 2. This trend was also observed at the 9-month point, with a marginally significant difference (*p* = 0.055) in ΔHAZ between those with protozoa and those without at 12 months and significant (*p* = 0.046) for ΔHAZ at 24 months of age (Table 3).

**TABLE 2:** Association of enteropathogens at 3–6 months (*n* = 416) with change in height-for-age (ΔHAZ), weight-for-age z-scores (ΔWAZ), and weight-for-height z-scores (ΔWHZ) reported as median (Q1, Q3) during first (12 months) and the second year of life (24 months).

	Bacteria			Protozoa			Viruses		
	No	Yes	*p*	No	Yes	*p*	No	Yes	*p*
*N* (%)	104 (25.00%)	312 (75.00%)		205 (49.30%)	211 (50.70%)		217 (52.20%)	199 (47.80%)	
ΔHAZ 12 months	−0.50 (−1.31, 0.17)	−0.57 (−1.47, 0.10)	0.690	−0.40 (−1.22, 0.16)	−0.75 (−1.65, −0.03)	**0.011**	−0.52 (−1.27, 0.16)	−0.57 (−1.54, 0.07)	0.400
ΔHAZ 24 months	−0.53 (−1.30, −0.14)	−0.74 (−1.49, 0.05)	0.700	−0.43 (−1.25, 0.17)	−0.90 (−1.64, −0.10)	**0.004**	−0.57 (−1.37, 0.05)	−0.88 (−1.53, −0.01)	0.340
ΔWAZ 12 months	−0.48 (−1.14, 0.53)	−0.50 (−1.26, 0.44)	0.990	−0.49 (−1.11, 0.36)	−0.50 (−1.37, 0.49)	0.490	−0.56 (−1.12, 0.49)	−0.46 (−1.30, 0.44)	0.980
ΔWAZ 24 months	−0.36 (−1.04, 0.55)	−0.44 (−1.08, 0.46)	0.910	−0.40 (−1.04, 0.55)	−0.44 (−1.15, 0.46)	0.450	−0.37 (−1.08, 0.54)	−0.44 (−1.06, 0.44)	0.990
ΔWHZ 12 months	−0.07 (−0.91, 0.53)	−0.36 (−1.24, 0.67)	0.780	−0.37 (−1.22, 0.56)	−0.34 (−1.10, 0.65)	0.970	−0.51 (−1.24, 0.51)	−0.26 (−1.00, 0.80)	0.240
ΔWHZ 24 months	0.05 (−0.74, 0.64)	−0.10 (−0.74, 0.72)	0.770	−0.05 (−0.75, 0.84)	−0.07 (−0.71, 0.67)	0.970	−0.07 (−0.70, 0.51)	−0.08 (−0.75, 1.02)	0.410

Data are expressed as median (Q1, Q3); Q1: 1st quartile; Q3: 3rd quartile.

“Yes” = infection with at least one or more pathogen of the specific category.

“No” = no infection of the specific category.

ΔHAZ, WAZ, and WHZ 12 months = change in the HAZ, WAZ, and WHZ scores from baseline to end of the first year of life.

ΔHAZ, WAZ, and WHZ 24 months = change in the HAZ, WAZ, and WHZ scores from baseline to end of the second year of life. Bold values show significant *p*-values.

**TABLE 3:** Association of enteropathogens at 9 months (*n* = 416) with change in height-for-age (ΔHAZ), weight-for-age z-scores (ΔWAZ), and weight-for-height z-scores (ΔWHZ) reported as median (Q1, Q3) during first (12 months) and the second year of life (24 months).

	Bacteria			Protozoa			Viruses		
	No	Yes	*p*	No	Yes	*p*	No	Yes	*p*
*N* (%)	89 (21.40%)	327 (78.60%)		171 (41.10%)	245 (58.90%)		194 (46.60%)	222 (53.40%)	
ΔHAZ 12 months	−0.50 (−1.01, −0.09)	−0.56 (−1.49, 0.12)	0.350	−0.45 (−1.24, 0.32)	−0.61 (−1.55, 0.00)	**0.055**	−0.49 (−1.23, 0.09)	−0.58 (−1.65, 0.10)	0.160
ΔHAZ 24 months	−0.42 (−0.90, −0.25)	−0.76 (−1.50, 0.05)	0.570	−0.51 (−1.28, 0.14)	−0.83 (−1.55, −0.03)	**0.046**	−0.53 (−1.30, −0.04)	−0.84 (−1.62, 0.10)	0.320
ΔWAZ 12 months	−0.25 (−1.01, 0.70)	−0.51 (−1.26, 0.43)	0.360	−0.52 (−1.09, 0.53)	−0.42 (−1.30, 0.44)	0.550	−0.38 (−1.05, 0.44)	−0.54 (−1.39, 0.45)	0.320
ΔWAZ 24 months	−0.24 (−1.14, 0.41)	−0.44 (−1.07, 0.52)	0.630	−0.34 (−1.14, 0.41)	−0.45 (−1.06, 0.53)	0.810	−0.26 (−1.03, 0.52)	−0.44 (−1.15, 0.46)	0.420
ΔWHZ 12 months	−0.08 (−1.11, 0.81)	−0.36 (−1.16, 0.58)	0.550	−0.38 (−1.11, 0.62)	−0.33 (−1.24, 0.64)	0.870	−0.26 (−1.22, 0.73)	−0.41 (−1.10, 0.56)	0.550
ΔWHZ 24 months	0.09 (−0.62, 0.82)	−0.09 (−0.74, 0.71)	0.640	−0.04 (−0.75, 0.76)	−0.08 (−0.69, 0.71)	0.850	0.07 (−0.60, 0.81)	−0.14 (−0.77, 0.65)	0.250

Data are expressed as median (Q1, Q3); Q1: 1st quartile; Q3: 3rd quartile.

“Yes” = infection with at least one or more pathogen of the specific category.

“No” = no infection of the specific category.

ΔHAZ, WAZ, and WHZ 12 months = change in the HAZ, WAZ, and WHZ scores from baseline to end of the first year of life.

ΔHAZ, WAZ, and WHZ 24 months = change in the HAZ, WAZ, and WHZ scores from baseline to end of the second year of life. Bold values show significant *p*-values

### The burden of enteropathogen in fecal samples

**FIGURE 1:**
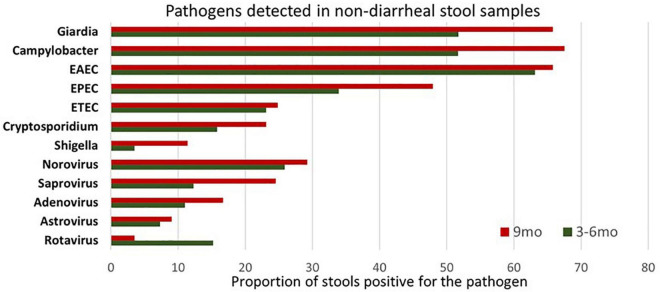
Illustration of enteropathogen burden in surveillance stool samples at 3–6 months and 9 months’ time point. Pathogens considered positive/negative based on Ct value cut-off. EAEC burden was highest at 3–6 months of age while Giardia and Campylobacter at 9 months. Enteroaggregative *Escherichia coli* (EAEC) Enteropathogenic *Escherichia coli* (EPEC) Enterotoxigenic *Escherichia coli* (ETEC).

Next, we explored the presence of individual pathogens in the fecal samples at 6 and 9 months. No pathogen was detected in 83/416 and 80/416 samples at 3–6 months and at 9 months, respectively. As expected, *Giardia*, *Campylobacter* spp., EAEC, EPEC, ETEC, and *Cryptosporidium* spp. were the most prevailing enteropathogens in terms of overall positivity at both time points (Figure 1). Amongst viruses, Norovirus and sapovirus were found in more than 25% of samples at both time points. At the same time, detection of rotavirus dropped by 9 months, as explained by the rotavirus vaccine that was administered to these children at the time of enrollment and stool sample collection (3–6 months of age). Regarding persistent infection, pathogens that reported positive at both time points included 143 (34.38%) *Giardia*, 188 (45.19%) *Campylobacter* spp., 41 (9.86%) ETEC, 13 (3.13%) *Shigella* spp. and 19 (4.57%) *Cryptosporidium*.

### Individual pathogens colonization is associated with growth restriction

Next, we selected pathogens based on prevalence and explored their association with growth velocity. As reported in Tables 4, 5, a negative trend was seen on ΔHAZ in children with *Giardia* in their stools at 3–6 months and 9 months. This association was observed with growth velocity during both years, yet a stronger effect was seen on ΔHAZ 24 months. No difference was seen in the diarrheal episodes in children with or without *Giardia* (*p* = 0.15). On the other hand, children who had *Campylobacter* spp. in their non-diarrheal stool samples had a significantly higher number of diarrheal episodes per year.

**TABLE 4:** Individual pathogens colonization is associated with growth restriction at the time of enrollment in the study (3–6 months).

	Selected enteropathogen at 3–6 months											
	*Giardia*			*Campylobacter* spp.			ETC			*Cryptosporidium* spp.		
	No	Yes	*p*	No	Yes	*p*	No	Yes	*p*	No	Yes	*p*
*N* (%)	165 (48.20%)	177 (51.80%)		190 (45.70%)	226 (54.30%)		322 (77.40%)	94 (22.60%)		288 (84.20%)	54 (15.80%)	
ΔHAZ 12 months	−0.49 (−1.30, 0.15)	−0.67 (−1.60, 0.08)	0.130	−0.44 (−1.27, 0.17)	−0.64 (−1.52, 0.07)	0.160	−0.53 (−1.48, 0.15)	−0.67 (−1.41, 0.07)	1.000	−0.50 (−1.35, 0.17)	−0.91 (−1.83, −0.24)	0.027
ΔHAZ 24 months	−0.54 (−1.29, 0.22)	−0.88 (−1.67, −0.12)	**0.012**	−0.51 (−1.30, 0.04)	−0.85 (−1.53, 0.05)	0.140	−0.63 (−1.48, 0.04)	−0.78 (−1.46, 0.01)	0.970	−0.64 (−1.40, 0.10)	−0.97 (−1.69, −0.30)	0.051
ΔWAZ 12 months	−0.51 (−1.14, 0.29)	−0.42 (−1.29, 0.58)	0.950	−0.35 (−1.14, 0.59)	−0.55 (−1.3, 0.30)	0.200	−0.45 (−1.14, 0.43)	−0.55 (−1.40, 0.46)	0.560	−0.45 (−1.20, 0.56)	−0.50 (−1.26, 0.05)	0.560
ΔWAZ 24 months	−0.42 (−1.04, 0.53)	−0.39 (−1.11, 0.53)	0.870	−0.23 (−1.04, 0.55)	−0.47 (−1.12, 0.46)	0.370	−0.42 (−1.06, 0.51)	−0.50 (−1.11, 0.46)	0.850	−0.41 (−1.05, 0.55)	−0.53 (−1.10, 0.21)	0.440
Diarrhea episodes/year	12.00 (8.0, 18.0)	14.0 (10.0, 19.0)	0.150	11.0 (6.0, 17.0)	14.0 (9.0, 19.0)	**0.004**	13.0 (7.0, 18.0)	13.0 (8.0, 18.0)	0.410	14.0 (8.0, 19.0)	14.0 (10.0, 18.0)	0.600

Data are expressed as Median (Q1, Q3); Q1: 1st quartile; Q3: 3rd quartile. “Yes” = infection with one or more pathogen of the specified spp. “No” = no infection of the specified pathogen. Bold values show significant *p*-values.

**TABLE 5:** Individual pathogens colonization is associated with growth restriction at 9 months of age.

	Selected enteropathogen at 9 months											
	*Giardia*			*Campylobacter* spp.			ETC			*Cryptosporidium* spp.		
	No	Yes	*p*	No	Yes	*p*	No	Yes	*p*	No	Yes	*p*
*N* (%)	117 (34.20%)	225 (65.80%)		141 (33.90%)	275 (66.10%)		299 (71.90%)	117 (28.10%)		263 (76.90%)	79 (23.10%)	
ΔHAZ 12 months	−0.42 (−1.22, 0.34)	−0.73 (−1.60, −0.03)	**0.031**	−0.23 (−1.15, 0.36)	−0.67 (−1.57, −0.04)	**0.005**	−0.50 (−1.31, −0.04)	−0.70 (−1.61, 0.18)	0.520	−0.49 (−1.42, 0.18)	−0.95 (−1.65, −0.24)	**0.015**
ΔHAZ 24 months	−0.52 (−1.28, 0.33)	−0.88 (−1.62, −0.08)	**0.006**	−0.38 (−1.02, 0.19)	−0.90 (−1.61, −0.08)	**0.001**	−0.74 (−1.46, 0.00)	−0.63 (−1.53, 0.20)	0.850	−0.60 (−1.46, 0.18)	−0.92 (−1.50, −0.35)	**0.016**
ΔWAZ 12 months	−0.51 (−1.06, 0.56)	−0.46 (−1.39, 0.44)	0.220	−0.12 (−0.87, 0.83)	−0.61 (−1.34, 0.29)	**0.002**	−0.52 (−1.20, 0.39)	−0.34 (−1.27, 0.57)	0.500	−0.43 (−1.27, 0.55)	−0.62 (−1.18, 0.31)	0.480
ΔWAZ 24 months	−0.23 (−1.01, 0.57)	−0.50 (−1.07, 0.46)	0.130	−0.10 (−0.88, 0.62)	−0.50 (−1.15, 0.35)	**0.013**	−0.44 (−1.11, 0.41)	−0.32 (−1.04, 0.61)	0.480	−0.35 (−1.05, 0.54)	−0.55 (−1.07, 0.41)	0.450
Diarrhea episodes/year	13.00 (9.00, 18.00)	14.00 (9.00, 19.00)	0.370	11.00 (3.00, 17.00)	13.00 (8.00, 19.00)	**0.001**	12.00 (6.00, 17.00)	14.00 (10.00, 19.00)	**<0.001**	13.00 (8.00, 19.00)	14.00 (9.00, 19.00)	0.450

Summarized using Median (Q1, Q3); Q1: 1st quartile; Q3: 3rd quartile. “Yes” = infection with one or more pathogen of the specified spp. “No” = no infection of the specified pathogen. Bold values show significant *p*-values.

Regarding its effect on growth velocity, no difference was seen in the presence or absence of *Campylobacter* spp. at 3–6 months, however, detection at 9 months led to a significant drop in ΔHAZ and ΔWAZ from baseline to end of the first year (12 months) and even from baseline to end of the second year of life (Table 5). Detection of ETEC did not affect the growth velocity and only reported an association with a higher number of diarrheal episodes when detected at 9 months (*p* < 0.001).

### Exploration of co-pathogen colonization and their association with growth faltering

**FIGURE 2:**
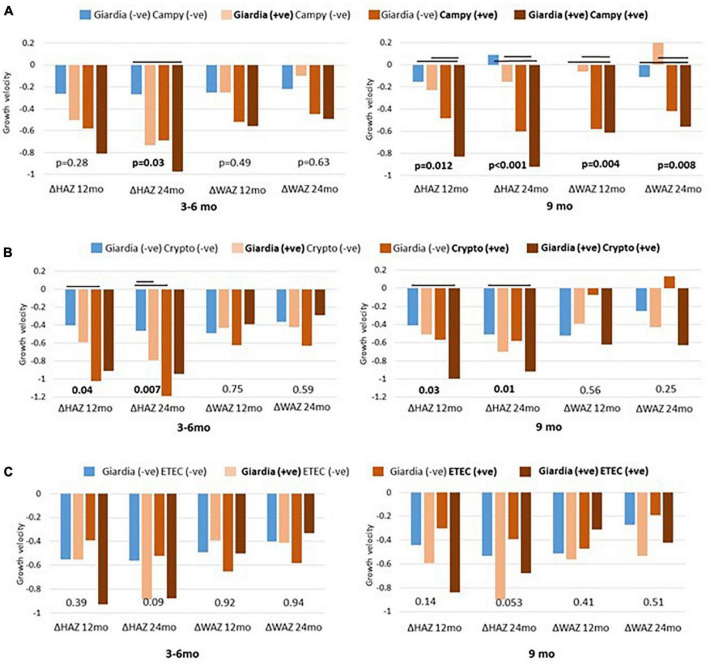
Co-pathogen colonization and their association with growth matrices. Co-existence of giardia and campylobacter (Campy) plotted as **(A)** giardia and cryptosporidium (Crypto) as **(B)** and giardia and enterotoxigenic *Escherichia coli* (ETEC) as **(C)**. Significant differences (*p* < 0.05) are marked as black bars between the groups at 3 to 6 and 9 months’ time point.

**FIGURE 3:**
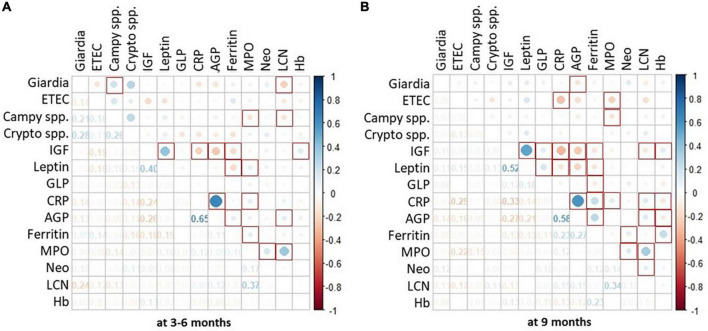
Association of enteropathogen colonization with systemic and fecal biomarkers. Heat map shows burden of enteropathogen as the size of the dot whereas the color reports positive or negative association with serum and fecal biomarkers. Part **(A)** of the figure depicts data from 1st time point i.e., 3–6 months. Whereas, part **(B)** depicts data from 2nd time point i.e., 9 months. *P*-value < 0.05 is considered significant and marked as red squares. *Spearman correlation using quantitative values (Ct) of pathogens detected on TAC and quantitative values for biomarkers.

To gain further insight into the association of *Giardia* detection with growth velocity, we explored the role of co-pathogen colonization. A significant agreement was observed between the presence of *Giardia* at 9 months with the presence of *Campylobacter* spp. (*p* < 0.000), *Cryptosporidium* spp. (*p* = 0.003) and ETEC (*p* = 0.004). Hence, we further explored the simultaneous detection of *Giardia* with these pathogens. Co-colonization of *Giardia* with *Campylobacter* spp. at 9 months significantly hindered with linear and ponderal growth velocity (ΔHAZ, ΔWAZ) during both years (Figure 2) while their simultaneous detection at 3–6 months significantly negatively impacted ΔHAZ at 24 months. On the other hand, co-colonization of *Giardia* and *Cryptosporidium* early in life seemed to have a protective role as children with both the pathogens had a better growth velocity than those with only *Cryptosporidium* (Figure 2B). This trend was significant for ΔHAZ while non-significant for ΔWAZ; however, it was lost by 9 months. No association was found regarding the co-colonization of *Giardia* with ETEC (Figure 2C). We further explored the enigmatic association of biomarkers and enteropathogens using quantitative Spearman correlation. A significant positive association was seen in Giardia with LCN (*p* = 0.001) at 3–6 months of age. Whereas with AGP (*p* = 0.03) at 9 months of age. Campylobacter spp. also showed a significant positive association with MPO (*p* = 0.03) and LCN (*p* = 0.04) at 3–6 months and with MPO (*p* = 0.01) at 9 months (Figure 3).

## Discussion

This study estimates the magnitude of enteropathogen burden in non-diarrheal stools and explores the association with fecal biomarkers in children living in EED endemic settings. Protozoa reported the most significant impact on the linear growth velocity, amongst which *Giardia* colonization during the early months of life was associated with growth faltering. The cumulative effect of other enteropathogens such as *Campylobacter* spp. and *Cryptosporidium* spp. further impeded the growth. As EED is a subclinical condition, enteropathogen detection in non-diarrheal stool and its association with growth faltering is consistent with previous work that shows pathogens’ effect on young children’s growth.

In our study, the most common pathogens detected included *Giardia*, *Campylobacter* spp., diarrheagenic *E. coli* spp., *Cryptosporidium* spp., and Norovirus. At least 50% of fecal samples had *Giardia* as early as 3–6 months of age, with even higher positivity (67%) by 9 months. *Giardia* colonization affected ΔHAZ by 24 months and was associated with concurrent colonization of *Campylobacter* spp., *Cryptosporidium* spp., and ETEC. A significant decline in ΔHAZ and ΔWAZ was noticed in children who were simultaneously positive for *Giardia* and *Campylobacter* spp. On the other hand, the simultaneous presence of *Giardia* and *Cryptosporidium* had a significant effect on ΔHAZ (for both years), while no effect was seen on ΔWAZ. Table 6 summarizes the most common pathogens reported in diarrheal and non-diarrheal stool samples collected from children living in LMICs.

**TABLE 6:** Most common enteropathogen identified in diarrheal/non-diarrheal stool samples from large-scale studies from LMICs.

	Population	Sample	Method	Findings
Donowitz et al. (3)	A longitudinal birth cohort study of 250 children from Bangladesh	Diarrheal stool samples	PCR	Rotavirus, *Campylobacter* spp., and *Shigella* spp. were the leading causes of diarrhea in the first year, while *Shigella*, *Campylobacter* spp., and ST-ETEC in the second year of life. Only Norovirus reported an association with 24 months LAZ and neurodevelopment.
Schnee et al. (27)	Bangladesh	Diarrheal stools and blood samples	CRP ELISA, Stool TAC	*Campylobacter* spp. were associated with the highest attributable incidence, followed by rotavirus, adenovirus, *Shigella*/EIEC, STEC, and ETEC.
MAL-ED study, Platts-Mills et al. (28)	Bangladesh, India, Nepal, Pakistan, South Africa, Brazil, Tanzania, Peru	Diarrheal and non-diarrheal stools	Stool TAC	Subclinical infections, particularly *Shigella*, EAEC, *Campylobacter* spp. and *Giardia* were negatively associated with linear growth during the first 2 years of life and sometimes up to 5 years.
MAL-ED study, Platts-Mills et al. (6) and Rogawski et al. (29)	Birth cohort data of Bangladesh	Diarrheal stool and blood samples	ELISA and qPCR	*Giardiasis*, Ascariasis, and Trichuriasis were the most frequent parasitic infections, while *Campylobacter* spp., EAEC, and ETEC were the common bacterial pathogens.
Rogawski et al. (15)	Bangladesh, Brazil, India, Nepal, Pakistan, Peru, South Africa, Tanzania	Non-diarrheal and diarrheal stool samples	Enzyme immunoassay	Persistent *Giardiasis* was detected in 40% of the children. Independent of diarrhea, *Giardia*sis might contribute to intestinal permeability and stunted growth.
Lima et al. (30)	Brazil	Non-diarrheal stool sample		The top five most prevalent enteric pathogens were atypical EPEC, EIEC, *Giardia* spp., EAEC, and *Campylobacter* spp. EAEC was more prevalent in malnourished children.
GEMS study, Liu et al. (31)	Bangladesh, India, Pakistan, Gambia, Kenya, Mali, and Mozambique	Diarrheal and non-diarrheal stools	Stool TAC	The six most attributable pathogens were *Shigella* spp., rotavirus, adenovirus, ST-ETEC, *Cryptosporidium* spp., and *Campylobacter* spp. These pathogens accounted for 77⋅8% of all attributable diarrhea.

Based on single pathogen colonization data, contradictory findings supporting the protective role of *Giardia*, especially in early childhood have been reported. In the multicenter MAL-ED study, only the Pakistani site reported an association of persistent *Giardia* detection with a relative decrease in diarrheal rates (15). In a Bangladeshi study, early life *Giardia* colonization increased the risk of stunting at 2 years of age but not for poor weight gain as it neither increases nor decreases the odds of acute all-cause diarrheas (16). Experiments by Luther et al. using protein-deficient mice demonstrate that microbial adaptations to undernutrition combined with cumulative enteropathogen influence host growth, gut immune response, and metabolism (17). Using *Giardia lamblia* and enteroaggregative *Escherichia coli* (EAEC) co-colonization, *Giardia* overcame microbiota-mediated pathogen clearance during protein malnutrition, promoting growth impairment and alteration of small intestinal 16S abundance and mucosal immunity that converges with the metabolic responses to worsen host growth. Their data model the effect of early life cumulative enteropathogen exposures on disruption of intestinal immunity and host metabolism during crucial developmental periods. We observed similar trends in our study cohort where *Cryptosporidium* spp. detection in the presence of *Giardia* before 6 months had minimal impact on the growth velocity, yet this protection was lost at 9 months. As intestinal microbiota develops during the early months of life, this protection might provide the critical window of susceptibility. Hence, further studies are required on the role of gut microbiota and enteropathogen co-colonization in undernourished children to gain insight into the pathophysiology of EED. In the context of stunting and malnutrition, our group has already reported increased intestinal permeability in children with *Giardia* detected in their duodenal aspirates, even in the absence of chronic inflammation of the gut tissue (15, 18). *Giardia*sis has shown a minimal association with markers of intestinal inflammation, further suggesting a non-inflammatory mechanism (19).

*Campylobacter* spp. infection is a gastrointestinal bacterial infection widespread in low- and middle-income countries. In urban Bangladeshi participants of the MAL-ED study, a negative impact was reported on linear growth in children between 12 and 21 months of age (20). In another prospective birth cohort of 271 Peru children, disruption to the fecal microbiota seems to explain the effects of Campylobacter infection on growth (21). We also observed a significant decline in the growth velocity (height and weight) of children who were positive for *Campylobacter* spp. in the presence of *Giardia* at 9 months; however, we did not explore their fecal microbiota. Regarding *Cryptosporidium* detection, co-colonization with *Giardia* was significantly associated with linear growth velocity, although these samples were non-diarrheal. Cryptosporidiosis is a well-known risk factor for diarrhea-associated morbidity and mortality in regions with contaminated water (22). However, it has also been reported in non-diarrheal stool samples (23). With the advent of the propagation of *Cryptosporidium* in organoids, exploring the pathophysiology of co-infections, especially in the context of malnutrition, may provide avenues of intervention (24).

Additionally, we explored the association of serum and fecal biomarkers with the selected pathogen. At 3–6 months, fecal lipocalin reported a significant association with *Giardia* and *Campylobacter* spp. yet this trend was lost at 9 months. On the other hand, a consistent association between fecal MPO and *Campylobacter* spp. was seen at both time points. Due to the complex pathophysiology of EED, serum and fecal biomarkers of intestinal inflammation have shown an inconsistent association. Undernutrition increases the risk of under-development of the immune system during infant life, coupled with environmental toxins and pathogen exposure *via* the oral-fecal route. Hence, efforts to utilize inflammatory biomarkers for diagnosing and monitoring the progression of EED have rendered mixed results. Lipocalin-2 is a bacteriostatic peptide that prevents bacterial growth by hindering iron uptake of the pathogens (25). Various studies have assessed lipocalin-2 as a marker of inflammation in a diseased state. Our group has observed higher lipocalin-2 expression in the duodenal tissue collected from children at risk of developing EED (9). Diarrheal pathogens have correlated with known serum and fecal inflammatory biomarkers; however, their utility in diagnosing EED has not been established (7).

Finally, we determined the distribution of the *ctxM*, *gyrA*, and *parC* genes to explore antimicrobial resistance genes. At least one of these AMR genes was detected in more than 75% of samples by 9 months of age, with the highest prevalence of *ctxM* gene, which correlates with the exuberant use of β-lactam antibiotics. Undernourished children are already at a higher risk of infectious diseases due to weakened immune system, and the additional burden of AMR genes expose them to even worse outcomes. This may explain why antibiotic trials to mitigate the effects of enteropathogen exposure on growth faltering have failed to produce desirable results (26).

Limitations of this study include a collection of samples at two-time points only. Moreover, although we strictly collected non-diarrheal samples, data about antibiotics received by children during that month was not available that might have influenced the presence or absence of pathogens. In cases of active diarrhea, sample collection was delayed for at least a week. As a result, this study does not report the burden of the pathogen in the diarrheal stool focusing on improving our understanding of the role of asymptomatic enteropathogen colonization in the context of EED. Although we designed this study to recruit children as controls (healthy anthropometric measurements) along with cases, the anthropometric measurement of our controls regressed to the mean with time. Therefore, we could not explore the burden of enteropathogens in children with healthy growth trajectories living in similar settings. Lastly, we did not examine the fecal microbiota and its association with enteropathogen in linear and ponderal growth velocity.

## Conclusion

In conclusion, asymptomatic enteropathogen colonization with an increasing burden of pathogens, specifically protozoa, is associated with a decline in linear growth velocity during the first 2 years of life in children living in EED endemic settings. *Giardia* colonization is linked to simultaneous detection of pathogens such as *Campylobacter* spp. *Cryptosporidium* spp. and *ETEC*. Co-colonization with *Campylobacter* spp. had a detrimental effect on the linear growth velocity, while *Cryptosporidium* spp. detection in the presence of *Giardia* before 6 months had minimal impact on the growth velocity, yet this protection was lost at 9 months. We report the association of quantitative burden of *Giardia, Campylobacter* spp., and *ETEC* with fecal biomarkers such as lipocalin, myeloperoxidase, and serum AGP as well as CRP. Further studies are required to explore the role of cumulative microbial colonization, their adaptations to undernutrition, and their influence on gut homeostasis to unleash the link between asymptomatic enteropathogen burden and growth faltering in young children.

## Data availability statement

The raw data supporting the conclusions of this article will be made available by the authors, without undue reservation.

## Ethics statement

The studies involving human participants were reviewed and approved by the Ethical Review Committee (ERC# 3836-Ped-ERC-2015) of The Aga Khan University. Written informed consent to participate in this study was provided by the participants’ legal guardian/next of kin.

## Author contributions

AA and SRM: study conception and design. SA, FU, KS, FA, AK, SM, and AH: data collection. FK, ZJ, NI, IM, JM, SS, SRM, and AA: analysis and interpretation of results. FK, JI, and ZJ: draft manuscript preparation. All authors reviewed the results and approved the final version of the manuscript.
