# Supplementary material for: Impact of enteropathogens on faltering growth in a resource-limited setting
Source: Front Nutr. 2023 Jan 10;9:1081833. doi: 10.3389/fnut.2022.1081833 (PMC9871909; doi:10.3389/fnut.2022.1081833)
Supplement: Supplementary file 1 [file Table_1.docx]

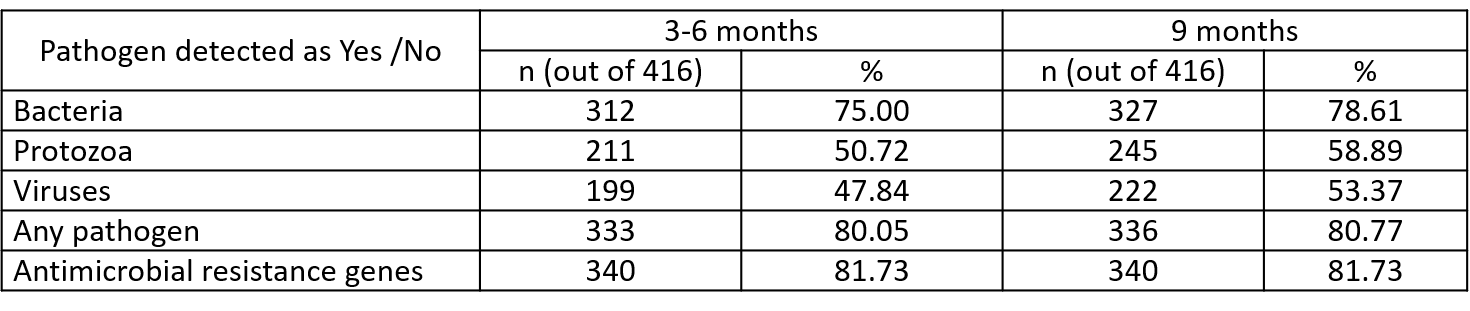


**Supplementary Table1:** Presence of enteropathogen and antimicrobial genes detected via TAC card based on Ct value cut-off.
